# Impact of NSAIDs and endurance exercise on myocardial fibrosis and arrhythmogenesis in murine coxsackieviral myocarditis

**DOI:** 10.1038/s41598-025-13437-x

**Published:** 2025-08-12

**Authors:** Sander Eens, Kasper Favere, Manon Van Hecke, Siel Van den Bogaert, Erik Fransen, Tania Roskams, Pieter-Jan Guns, Hein Heidbuchel

**Affiliations:** 1 Research Group Cardiovascular Diseases, GENCOR, University of Antwerp, Universiteitsplein 1, Antwerp, 2610 Belgium; 2 Laboratory of Physiopharmacology, GENCOR, University of Antwerp, Antwerp, 2610 Belgium; 3 Department of Cardiology, Antwerp University Hospital, Antwerp, 2650 Belgium; 4 Department of Cardiology, Ghent University, Ghent, 9000 Belgium; 5 Laboratory of Translational Cell and Tissue Research, Department of Imaging and Pathology, University of Leuven, Leuven, 3000 Belgium; 6 Centre of Medical Genetics, University of Antwerp and Antwerp University Hospital, Antwerp, 2610, 2650 Belgium

**Keywords:** Viral myocarditis, Physical exercise, Ibuprofen, Ventricular fibrosis, Arrhythmias, Cardiology, Medical research

## Abstract

Athletes often exhibit unexplained non-ischemic myocardial fibrosis, which is associated with malignant arrhythmias. Given the prevalent (over)use of NSAIDs among athletes and their harmful effects during viral myocarditis, this study examined the effects of combined NSAIDs and endurance exercise on the disease progression. To this end, male C57BL/6J mice underwent eight weeks of treadmill running (60 min/day; 18 cm/s) or no exercise. After two weeks, mice were implanted with mini-pumps delivering ibuprofen (70 mg/kg bw/day) or vehicle. Myocarditis was induced via intraperitoneal coxsackievirus inoculation. Mice were sacrificed six weeks post-inoculation for ventricular arrhythmogenicity evaluation and cardiac histopathological and molecular analysis. Exercising coxsackievirus-infected mice receiving ibuprofen recovered faster from weight loss. Mortality was low and similar across groups. Histopathology revealed abating inflammation and cell loss, without significant group differences. While exercise tended to increase extensive myocardial fibrosis, statistical analyses indicated no significant differences—with or without NSAIDs—in perivascular fibrosis, interstitial fibrosis, or myocardial scarring. NSAIDs—irrespective of exercise—did not increase arrhythmogenicity. In conclusion, ibuprofen in exercising mice with viral myocarditis resulted in faster weight loss recovery, without significant differences in inflammation, fibrosis, or arrhythmogenesis compared to exercise-only mice.

**Supplementary Information:**

The online version contains supplementary material available at 10.1038/s41598-025-13437-x.

## Introduction

Myocardial fibrosis is a hallmark of pathological cardiac remodeling, associated with cardiac dysfunction, malignant arrhythmias, and sudden cardiac death^1–3^. Recent cardiac magnetic resonance (CMR) imaging studies have revealed a frequent and unexplained occurrence of non-ischemic myocardial scarring among athletes with ventricular arrhythmias^4–6^. Notably, many of these athletes are young, otherwise healthy individuals with a low cardiovascular risk profile and no other cardiac abnormalities or identifiable genetic conditions. These observations underscore the urgent need for further investigation into the underlying pathophysiological mechanisms contributing to the increased prevalence of cardiac fibrosis in this population.

Epidemiological data, along with lesion locations and patterns, suggest that these myocardial scars may stem from prior (subclinical) myocarditis^2,7,8^. Notably, myocarditis can often occur “silently”, without clinically evident (viral or cardiac) symptoms^9,10^. It has been previously hypothesized that exercise itself could promote myocardial fibrogenesis during (subclinical) viral myocarditis^7^. In this context, in a mouse model of coxsackievirus B3-induced myocarditis, endurance exercise was shown to modulate the late-stage cardiac inflammatory response—with higher numbers of pro-inflammatory cells—and to enhance the development of extensive perivascular and interstitial fibrosis, along with a trend toward increased arrhythmogenesis^11^.

In this study, we evaluated whether non-steroidal anti-inflammatory drugs (NSAIDs) might synergistically contribute to this interaction. There is widespread use of NSAIDs among both recreational and professional athletes, often taken shortly before, during, or immediately after physical activity^12–14^. These drugs are typically used to manage musculoskeletal complaints or to alleviate symptoms of (potentially viral) infections. Animal studies have indicated that NSAIDs (e.g., ibuprofen, indomethacin, and sodium salicylate) can exacerbate myocardial inflammation and necrosis during the acute and subacute phases of viral myocarditis^15^. However, the long-term effects of NSAIDs in this context have not been explored. In this study, we focused on myocardial fibrosis and ventricular arrhythmogenesis in the long-term course of viral myocarditis in mice undergoing endurance exercise and receiving ibuprofen.

## Materials and methods

### Animals and ethical approval

A total of 104 male C57BL/6J wild-type mice (Charles River Laboratories, Belgium) were used in this study. The mice were housed in standard cages (a maximum of seven mice per cage) within the pathogenic unit at the University of Antwerp, under conventional housing conditions (12-hour light/dark cycle, 22 ± 2 °C, 40–65% relative humidity). All mice had ad libitum access to a standard chow diet and water, and experimental interventions were conducted during the light phase of the light/dark cycle. The study protocol was approved by the University of Antwerp Ethical Committee for Animal Testing (approval no. 2019-33) and adhered to the ARRIVE guidelines, the Belgian Royal Decree of 2013 (C – 2013/24,211) and the guidelines from the European Community Council Directive (2010/63/EU) concerning the protection of animals used for experimental purposes.

### Study design

At 15 weeks of age (D-14), mice were randomly assigned to one of four experimental groups (see Supplementary Fig. 1): NSAID-treated sedentary (CVB-NSAID-SED, *n* = 26), NSAID-treated exercised (CVB-NSAID-EEX, *n* = 26), and their corresponding vehicle-treated controls (CVB-SED, *n* = 26; CVB-EEX, *n* = 26). The exercise groups underwent an eight-week treadmill running protocol, starting two weeks before viral inoculation (D-14 to D0) and continuing for six weeks post-inoculation (D0 to D41). On D0, all mice were inoculated intraperitoneally with the human coxsackievirus B3 (CVB) strain Nancy to induce viral myocarditis. NSAIDs or vehicle were administered via subcutaneously implanted osmotic mini-pumps, providing continuous drug delivery for 28 days, beginning three days before viral inoculation (D-3 to D25). Most mice were sacrificed during the chronic disease phase (D41—45), while a smaller subset from each group (*n* = 4—6) was sacrificed during the acute phase (D7).

### Coxsackievirus B3 culture, quantification, and inoculation

The human coxsackievirus B3 strain Nancy was obtained from the American Tissue Culture Collection (France), cultured, and quantified using a plaque assay. Detailed information on virus culture and quantification is provided in previous work^11^. Animals were inoculated intraperitoneally in the lower right quadrant with 5 × 10^5^ plaque-forming units of virus, diluted in sterile phosphate-buffered saline (PBS) to a final injection volume of 500 µL per animal.

### Ibuprofen administration

Ibuprofen (I4883, Sigma-Aldrich, Germany) was administered through subcutaneously implanted osmotic mini-pumps (model 1004 W, RWD, China) designed for a sustained release over 28 days. Ibuprofen was dissolved in a 50:50 mixture of dimethyl sulfoxide (DMSO) and polyethylene glycol 300 (PEG300), providing an approximate daily dosage of 70 mg/kg body weight. The mini-pumps were implanted under anesthesia with 1.5–2.5% (v/v) isoflurane (AbbVie, Belgium) via a small incision in the upper back and were positioned toward the left flank. The incision was closed using sutures. Control animals received mini-pumps filled with the vehicle mixture only (i.e., DMSO and PEG300).

### Treadmill exercise training

Forced treadmill running was conducted using motorized treadmills equipped with an electric shock grid (LE8710MTS, Panlab, Spain). The exercise groups underwent treadmill running five days per week for eight consecutive weeks. Each session began with a 6-min warm-up: the first 3 min at 12 cm/s followed by 3 min at 15 cm/s. Next, the protocol extended for another 54 min at the target speed of 18 cm/s, resulting in a total session duration of 60 min. All exercise sessions were consistently conducted in the morning in a quiet environment, free from other animal species. To avoid any potential interference from the acute effects of the most recent exercise session, a 48-h rest period was implemented prior to sacrifice.

### Treadmill exhaustion testing

Treadmill exhaustion testing was conducted both at baseline, i.e., prior to the start of the study, and at the conclusion of the study. Incremental exercise testing commenced at a speed of 12 cm/s and was incrementally increased by 3 cm/s every 3 min until total exhaustion. Exhaustion was defined as the animal remaining for at least 5 s within the zone comprising the shock grid and one body length ahead of it. The treadmill inclination remained at 0° throughout the protocol. Prior to baseline testing, the animals were acclimated to treadmill running over three consecutive days at a walking speed of 5 cm/s. Before the second exhaustion test, the exercise groups underwent a 48-h period of exercise training abstinence.

### Cardiac electrophysiology study

Programmed electrical stimulation was performed via a transjugular right ventricular approach, following the previously established protocol from our group^16^. In brief, an octapolar catheter was positioned within the right ventricle. Subsequently, burst and ramp stimulation protocols were implemented to assess ventricular arrhythmogenicity. Detailed descriptions of these stimulation protocols are provided in the Supplementary Material. Arrhythmia inducibility and cumulative arrhythmia burden were assessed by considering only tachycardia episodes consisting of ≥ 3 consecutive ventricular beats, including both non-sustained (i.e., terminating spontaneously within 30 s) and sustained ventricular tachycardia (NSVT and VT, respectively). Arrhythmia inducibility refers to the ability to elicit ventricular arrhythmias and the specific step at which they are induced. The cumulative arrhythmia burden represents the total number of beats or the duration of these arrhythmias throughout the entire stimulation protocol.

### Euthanasia and tissue collection

Before sacrifice by exsanguination, animals were anesthetized with an intraperitoneal injection of sodium pentobarbital (150 mg/kg, Sanofi, Belgium). For cardiac electrophysiology testing, anesthesia was induced with Avertin^®^ (2,2,2-tribromoethanol, 250 mg/kg). Heparinized blood samples were centrifuged at 2000x*g* for 15 min at 4 °C to separate plasma. Immediately following dissection, hearts were immersed in ice-cold PBS containing 30 mmol/L potassium chloride to arrest the cardiomyocytes in diastole and remove residual blood and debris. The cardiac apex was then isolated, snap-frozen in liquid nitrogen, and stored at -80 °C for molecular work-up. The remaining heart tissue was fixed in a 4% neutral buffered formaldehyde for 24 h, followed by transfer to PBS until paraffin embedding and histological processing.

### Histology

Of each formalin-fixed and paraffin-embedded heart sample, two midventricular 5 μm sections were cut and stained with hematoxylin and eosin (HE; 3801540BBE and 3801590BBE, Leica Biosystems, Germany) or picrosirius red (PSR, without counterstain; 09400-25, Polysciences, Germany and 84512.260, VWR, USA). A trained and blinded pathologist evaluated myocardial inflammation and fibrosis using HE and PSR staining, respectively, employing an established, in-house semiquantitative scoring system^11^. In addition, quantitative assessments of inflammation and fibrosis were performed as previously described^11^. Cardiomyocyte cross-sectional area (CSA) was quantified to assess cellular enlargement. Cardiac tissue was stained with wheat germ agglutinin (WGA) to delineate cell membranes and 4′,6-diamidino-2-phenylindole (DAPI) to label nuclei. Fluorescent images were acquired using a Celena S fluorescence microscope, and CSA was measured using ImageJ software (version 1.54 g) by manually outlining individual cardiomyocytes. For each animal, CSA measurements were obtained from 40 cardiomyocytes, sampled from four randomly selected zones of a transverse tissue sections, with 10 cells analyzed per zone.

### Plasma ibuprofen concentrations

Ibuprofen plasma concentrations were determined as previously described^17^. The analysis was performed using a Trace GC Ultra coupled with a Trace DSQ MS detector (Thermo Fisher, USA).

A Restek RX2-5HT column was used with the following temperature gradient: 150 °C for 1 min, ramped at 20 °C/min to 220 °C and held for 1 min, then ramped at 10 °C/min to 300 °C and held for 1 min. The injector temperature was set at 250 °C with a split ratio of 1:10. For identification, the detector was operated in Total Ion Chromatogram (TIC) mode (50–200 m/z), while Selected Ion Monitoring (SIM) was used for quantification at 160 and 185 m/z.

A reference solution (RS) of 0.02 mg/ml ibuprofen (VWR, USA) in acetonitrile and an internal standard (IS) solution of 0.02 mg/ml naproxen (Roche, Switzerland) in acetonitrile were prepared. The reference solution was made by mixing 100 µl of IS stock, 100 µl of RS stock, 0.5 ml of water, and 0.5 ml of 85% phosphoric acid. For the test solution, 100 µl of IS stock and 0.5 ml of 85% phosphoric acid were added to 0.5 ml of plasma. To this, 4 ml of an ethyl acetate/hexane mixture (2:3, v/v) was added. This mixture was vortexed and then centrifuged at 8000 rpm for 10 min. The organic phase was dried under nitrogen and reconstituted in 100 µl of a 1:1 acetonitrile/BSTFA mixture, followed by vortexing. After a 10-min incubation, the sample was injected into the GC-MS system.

### RNA extraction

Total RNA was isolated from heart tissue using the RNeasy^®^ Fibrous Tissue Mini kit (74704, Qiagen GmbH, Germany), according to the manufacturer’s instructions. The concentration and purity of total RNA were evaluated by using the NanoDrop ND-2000 spectrophotometer (Thermo Fisher, USA).

### Digital PCR (dPCR)

Heart samples were randomly selected from all groups to quantify the viral load using the QIAcuity one-step viral RT-PCR kit, QIAcuity nanoplate 26k 24-well plates, QIAcuity One Digital PCR system, and the QIAcuity Software Suite (version 2.0.20), following the manufacturer’s instructions (Qiagen GmbH, Germany). The CVB3 genome was detected using the following primer-probe combination (IDT, Belgium):

forward primer 5’-GGTGCGAAGAGTCTATTGAGC-3’.

reverse primer 5’-CACCCAAAGTAGTCGGTTCC-3’.

probe 5’-/56- FAM/AATGCGGCT/ZEN/AATCCTAACTGCGGA/3IABkFQ/-3’.

### Quantitative PCR (qPCR)

Cardiac gene expression was quantified using a two-step qPCR approach with TaqMan™ Reverse Transcription Reagents (N8080234), TaqMan™ Universal PCR Master Mix (4304437), and Taqman™ primers (listed below), in accordance with the manufacturer’s instructions (Thermo Fisher, USA). For normalization, a reference gene combination comprising GAPDH (glyceraldehyde 3–8 phosphate dehydrogenase, Mm99999915_g1) and CDKN1B (cyclin-dependent kinase inhibitor 1b, Mm00438168_m1) was used. The following target genes were considered: CTGF (connective tissue growth factor, Mm01192933_g1), COL1A1 (collagen type I alpha 1 chain, Mm00801666_g1), COL3A1 (collagen type III alpha 1 chain, Mm0082305_g1), IL-1β (interleukin 1 beta, Mm00434228_m1), IL-6 (interleukin 6, Mm004446190_m1), and TNF-α (tumor necrosis factor alpha, Mm0043258_m1). The qPCR plates were run on a QuantStudio™ 3 instrument (Thermo Fisher, USA). Relative gene expression levels were calculated using the ∆∆CT method and reported as fold changes.

### Statistical analysis

The normality of the data was assessed visually and statistically using the Shapiro-Wilk test. Data are presented as mean ± standard error of the mean or as boxplots with median and 25th and 75th percentiles. Individual data points are displayed if applicable. Statistical analyses were performed using IBM SPSS Statistics v29.0.2.0 or GraphPad Prism v10.1.2, and graphs were generated using GraphPad Prism. Figures and figure legends provide details on the statistical tests, significance levels, and sample sizes for each analysis. The following significance levels were applied: *P < .05, **P < .01, ***P < .001, ****P < .0001.

## Results

### Disease course and survival

To investigate the effects of combined NSAID use and endurance exercise on the long-term course of viral myocarditis, mice underwent an eight-week treadmill running regimen while being implanted with mini-pumps for 28 days of ibuprofen delivery or a vehicle (see Supplementary Fig. 1). On D7 (i.e., after 10 days of ibuprofen administration), median plasma ibuprofen concentrations were 32.6 µg/ml in the CVB-NSAID-SED group and 24.0 µg/ml in the CVB-NSAID-EEX group (*P* = .762; data not shown). No detectable ibuprofen plasma levels were observed in either of the vehicle-treated groups.

**Fig. 1:**
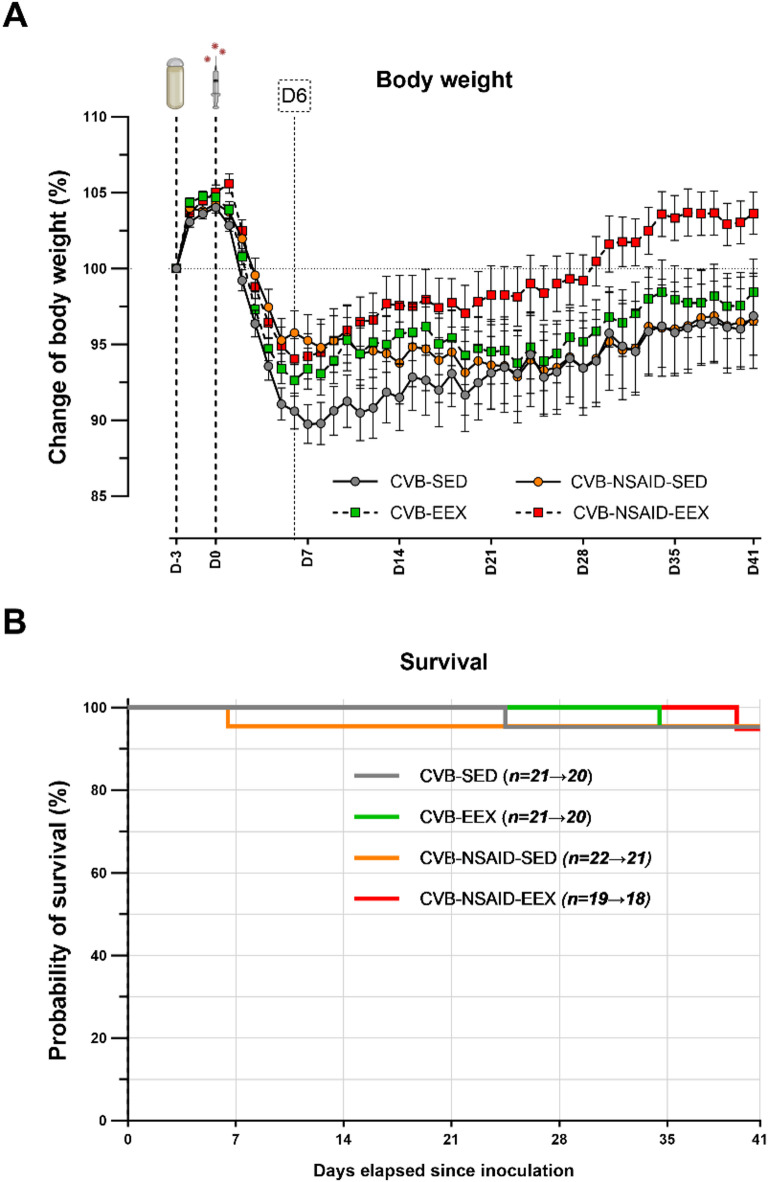
Body weight changes and survival. (**A**) Percentage change in body weight over the observation period. Data are presented as mean ± SEM. Piecewise linear regression models, with a ‘knot’ at day 6 to account for a change in slope, demonstrated significant differences in body weight changes between groups both before (*P* = .001) and after (*P* = .0001) the knot. (**B**) Kaplan-Meier survival analysis. Log-rank testing (Mantel-Cox) indicated no significant differences between groups (*P* > .999). Group sizes for panels (**A**,**B**) CVB-SED: *n* = 20, CVB-EEX: *n* = 20, CVB-NSAID-SED: *n* = 21, CVB-NSAID-EEX: *n* = 18.

Following viral inoculation, the mice exhibited rapid adverse health effects, including hunched posture, ruffled fur, and reduced spontaneous movement. Although these symptoms gradually improved over time, they persisted (partially) in the majority of animals until sacrifice at D41-45. Consistent with these observations, all groups experienced body weight loss during the first week post-inoculation, followed by gradual recovery (see Fig. 1A). All intervention groups experienced less pronounced acute weight loss (D0-D6) compared to the CVB-SED group, while the CVB-NSAID-EEX group demonstrated better long-term weight recovery (D6-D41).

Mortality was low and comparable across all groups (see Fig. 1B). All four groups experienced one premature death. The causes of these premature deaths are unclear; however, our historical data suggest they may be linked to severe gastrointestinal complications, such as pancreatitis^11,18^.

### Exercise training effects

The exercise groups followed a previously established treadmill protocol^11^. Training sessions were conducted at a target speed of 18 cm/s, which is estimated to correspond to approximately 75% of VO_2_ max^19^. One mouse in the CVB-NSAID-EEX group sustained a paw injury during the sessions and was therefore excluded from the study; all other mice successfully completed the protocol.

Functional assessments via exhaustion testing revealed no significant improvement in exercise capacity among the trained mice (see Supplementary Fig. 2A), consistent with what we previously reported for this strain^11^. While heart-to-body weight ratios and cardiomyocyte CSAs revealed no significant effects from the endurance exercise regimen itself, the cardiomyocyte CSA was significantly higher in the CVB-NSAID-EEX group, indicating cellular enlargement (e.g. hypertrophy)(see Supplementary Figs. 2B-C).

### Myocardial inflammation

**Fig. 2:**
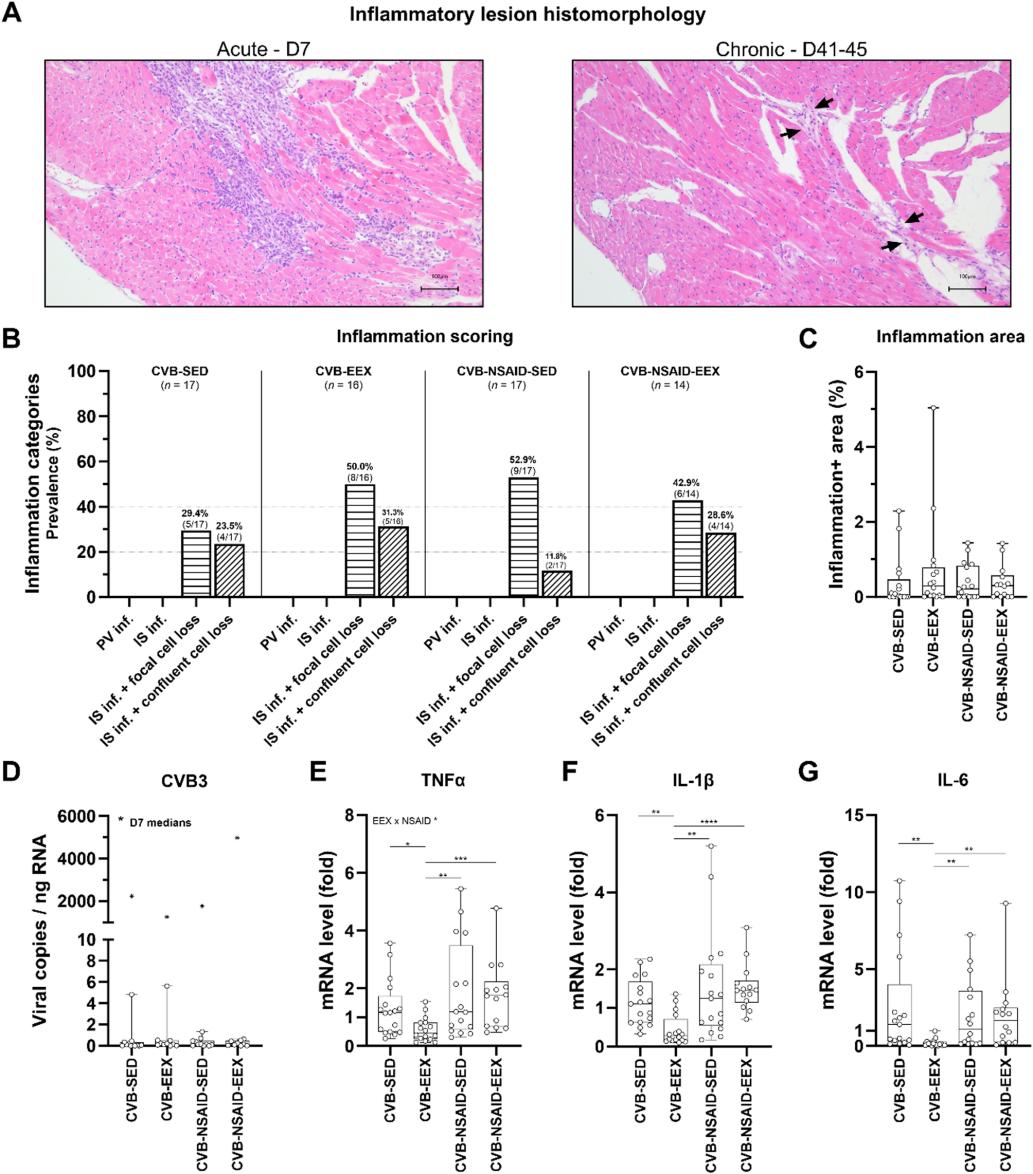
Myocardial inflammation at sacrifice. (**A**) Representative morphological images of the inflammatory lesions in the heart during the acute (D7) and chronic (D41-45) stages of coxsackievirus B3-induced myocarditis. Hematoxylin and eosin staining. Scale bar = 100 μm. (**B**) Semiquantitative scoring of myocardial inflammation. Monte Carlo chi-square testing indicated no significant differences between groups (*P* = .567). (**C**) Quantitative assessment of the area of inflammation in the myocardium. Kruskal-Wallis *H* testing revealed no significant differences between groups in the percentage of inflammation area (*P* = .493). (**D**) Cardiac CVB viral load at sacrifice was measured by dPCR. Kruskal Wallis *H* testing indicated no statistically significant differences between groups (*P* = .860). For reference, median viral load values from a subset of animals sacrificed 7 days post-inoculation are also included (*P* = .494). (**E–G**) Cardiac gene expression at sacrifice was assessed by qPCR. Messenger RNA levels are expressed as fold change relative to the CVB-SED group and normalized to GAPDH and CDK1NB. For TNFα, a two-way ANOVA with Tukey’s post-hoc test was performed, while statistical differences for IL-1β and IL-6 were evaluated using Kruskal-Wallis *H* testing followed by Dunn’s multiple comparisons. Data are presented as (**B**) frequency histograms or as (**C**–**G**) boxplots with interquartile ranges. Group sizes for panels (**B**–**G**) CVB-SED: *n* = 12–17, CVB-EEX: *n* = 10–16, CVB-NSAID-SED: *n* = 11–17, CVB-NSAID-EEX: *n* = 9–14.

Figure 2A shows representative images of inflammatory infiltrates observed during both the acute (D7) and chronic phases (D41-45) of the disease. In the acute phase, the cardiac phenotype was marked by significant inflammatory infiltration with confluent cell loss (see Supplementary Fig. 3). In the chronic phase, while inflammation had partially subsided, a substantial proportion of mice in all groups still showed interstitial inflammation along with either focal or confluent cell loss (see Fig. 2B). Analysis of myocardial inflammation scores during the chronic phase revealed no significant differences among the groups. Accordingly, the percentage of inflammatory area on cardiac cross-sections was similar across all groups at this phase (see Fig. 2C).

During the acute phase, median cardiac viral loads ranged from 1260 to 4973 copies/ng RNA across the groups. In contrast, during the chronic phase, viral material was nearly undetectable in all groups, with medians falling below 0.2 copies/ng RNA (see Fig. 2D), and one sample from each of the CVB-EEX and CVB-NSAID-SED groups tested virus-negative. For the inflammation-related genes TNF-α, IL-1β, and IL-6, expression levels were significantly reduced in the CVB-EEX group compared to all other groups (see Figs. 2E-G).

### Myocardial fibrosis

**Fig. 3:**
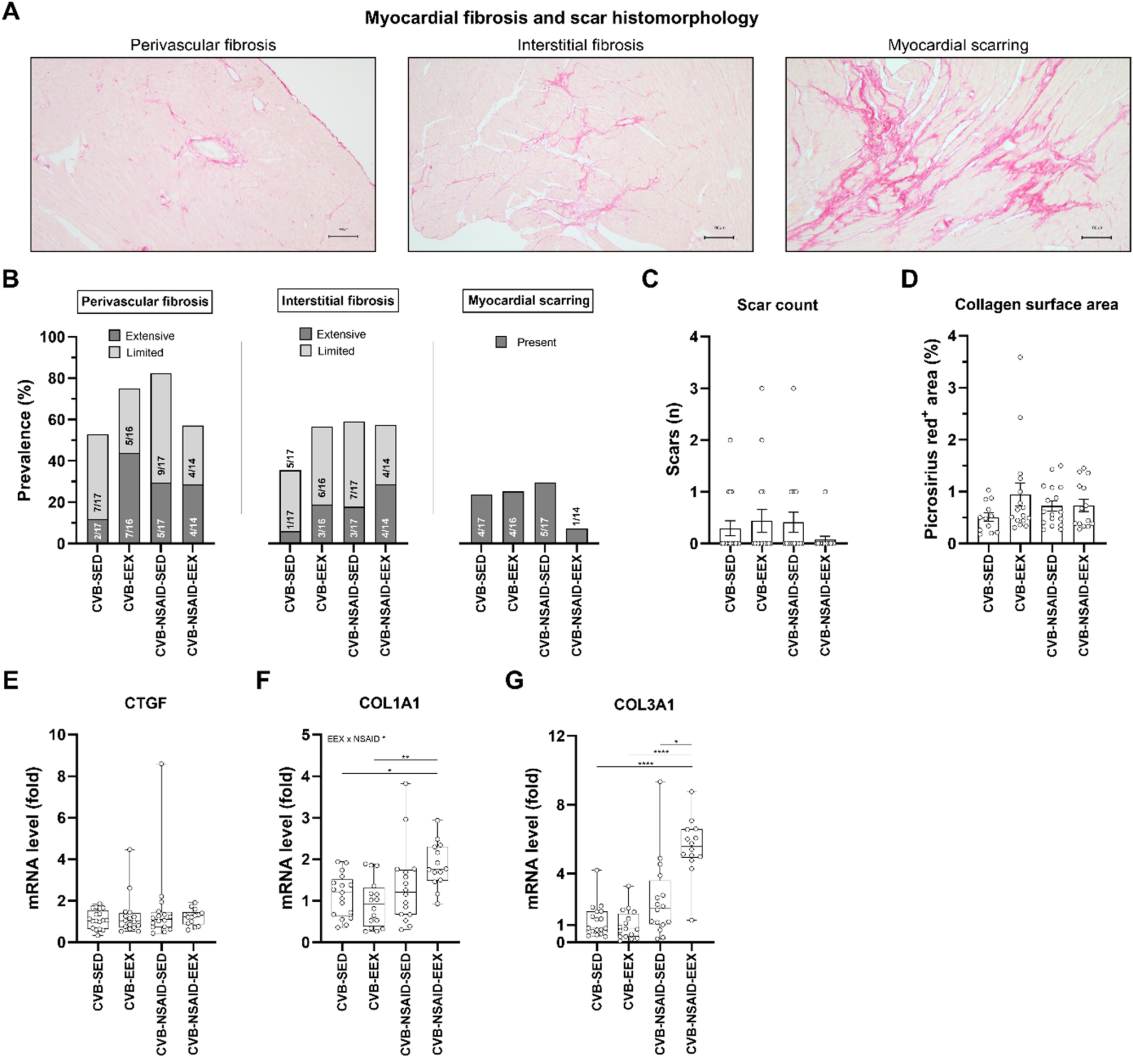
Myocardial fibrosis at sacrifice. (**A**) Representative morphological images of fibrotic lesions and scars in the heart during the chronic stage of coxsackievirus B3-induced myocarditis. Picrosirius red staining. Scale bar = 100 μm. (**B**) Semiquantitative scoring of myocardial fibrosis and scarring. Monte Carlo chi-square testing indicated no statistically significant differences in the extent of perivascular fibrosis (*P* = .273), interstitial fibrosis (*P* = .677), or myocardial scarring (*P* = .534) among the groups. (**C**) The myocardial scar counts and (**D**) collagen surface area, assessed by picrosirius red staining, were comparable between groups (*P* = .473 and *P* = .350, respectively). (**E**–**G**) Cardiac gene expression at sacrifice was assessed by qPCR. Messenger RNA levels are expressed as fold change relative to the CVB-SED group and normalized to GAPDH and CDK1NB. For genes CTGF and COL1A1, a two-way ANOVA with Tukey’s post-hoc test was performed, while statistical differences for COL3A1 were evaluated using Kruskal-Wallis *H* testing followed by Dunn’s multiple comparisons. Data are presented as (**B**) frequency histograms or as (**C**–**G**) boxplots with interquartile ranges. Group sizes for panels (**B**–**G**) CVB-SED: *n* = 12–17, CVB-EEX: *n* = 16, CVB-NSAID-SED: *n* = 17, CVB-NSAID-EEX: *n* = 14.

Figure 3A shows representative images illustrating perivascular fibrosis, interstitial fibrosis, and myocardial scarring during the chronic phase of the disease. Blinded histopathological analysis revealed the presence of both perivascular and interstitial fibrosis in all groups, with varying degrees of severity. Myocardial scarring was observed in only a subset of animals in each group, with prevalence rates ranging from 7.1 to 29.4% among the groups (see Figs. 3B-C). While we observed a trend toward more extensive fibrosis in exercising mice, consistent with our prior study^11^, statistical analyses indicated no significant differences in the extent of perivascular fibrosis, interstitial fibrosis, or myocardial scarring, with our without NSAIDs. Consistently, collagen surface areas, assessed via picrosirius red staining, were not significantly different across all groups (see Fig. 3D).

Figures 3E–G illustrate the effects on fibrotic markers. Cardiac CTGF mRNA levels were similar across all groups. In contrast, COL1A1 expression was significantly higher in the CVB-NSAID-EEX group compared to the vehicle-treated groups, and COL3A1 expression was significantly elevated in the CVB-NSAID-EEX group relative to all other groups.

### Ventricular arrhythmogenicity

**Fig. 4:**
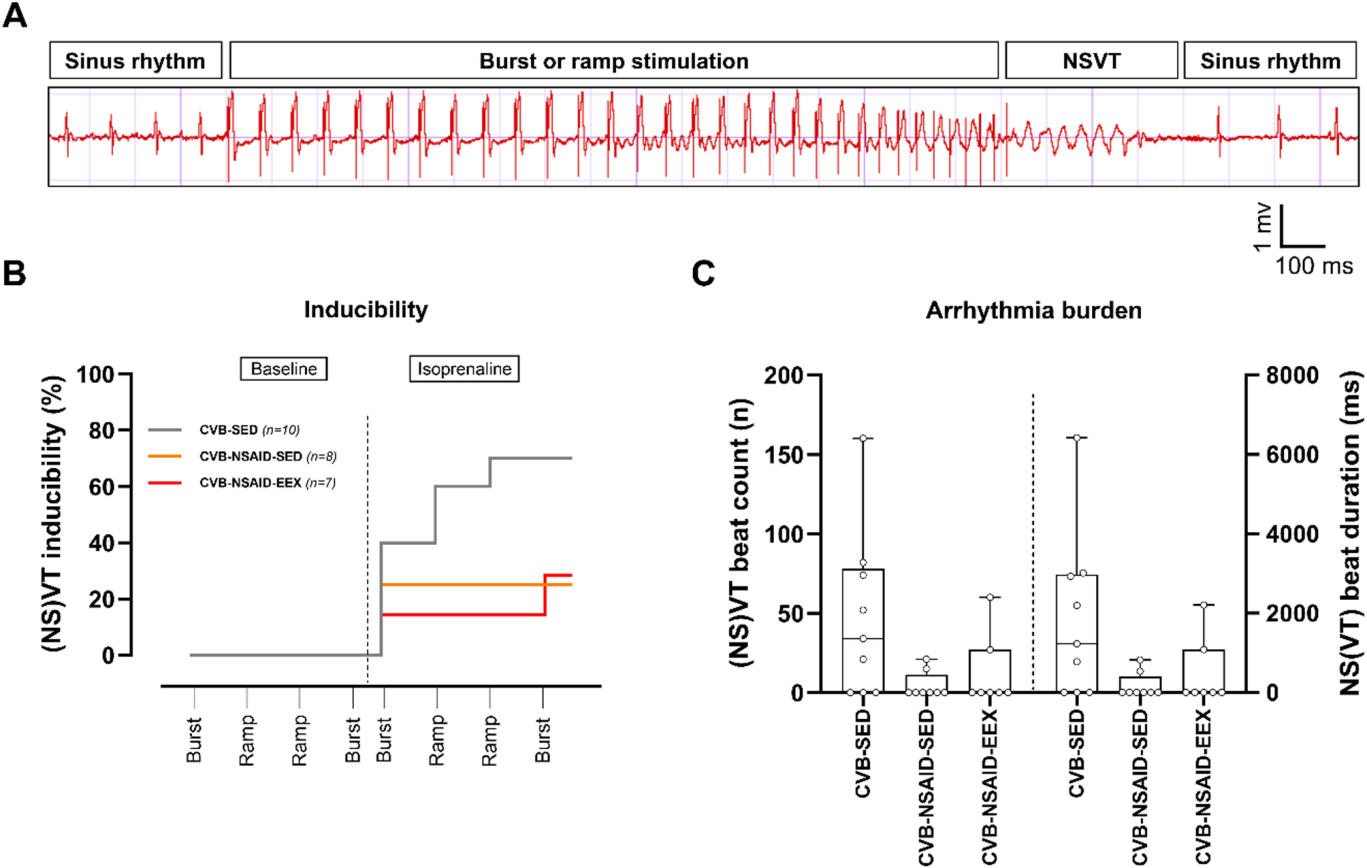
Ventricular arrhythmias immediately prior to sacrifice. (**A**) Representative illustration of a stimulus during programmed electrical stimulation, showcasing a NSVT episode. (**B**) Arrhythmia inducibility. Log-rank (Mantel-Cox) testing revealed no significant differences between groups (*P* = .096). (**C**) Cumulative arrhythmia burden, presented as both the number of beats and the duration in milliseconds. Kruskal-Wallis *H* testing with Dunn’s multiple comparisons indicated no statistically significant differences between groups for both the numbers of beats (*P* = .066) and duration (*P* = .071). Data are presented as boxplots with interquartile ranges. Group sizes for panels (**B**,**C**) CVB-SED: *n* = 9–10, CVB-NSAID-SED: *n* = 8, CVB-NSAID-EEX: *n* = 7.

Increasingly aggressive stimulation protocols were administered to a subset of the animals immediately before sacrifice, both at baseline and following the administration of the β-adrenoceptor agonist isoprenaline (see Fig. 4A). The CVB-EEX group was excluded from testing to maintain adequate sample sizes due to time constraints.

At baseline, no (NS)VT episodes were induced. After isoprenaline administration, ventricular arrhythmias were most frequently observed in the CVB-SED group, affecting 7 of out 10 mice (see Fig. 4B). In contrast, the CVB-NSAID-SED and CVB-NSAID-EEX groups had fewer occurrences, with 2 out of 8 and 2 out of 7 mice affected, respectively. These episodes predominantly occurred during the initial stages of the stimulation protocol and were characterized solely by non-sustained (typically polymorphic) runs.

Figure 4C illustrates the median cumulative duration of arrhythmia episodes throughout the stimulation protocol, presented as both the total number of beats and the total duration in milliseconds. The median arrhythmia burden in the CVB-SED group (34 beats, corresponding to 1227.3 ms) was higher compared to that in the NSAID-treated groups (0 beats/ 0 ms for both), although this difference was not statistically significant.

## Discussion

Recent studies have highlighted a significant prevalence of enigmatic non-ischemic myocardial fibrosis in athletes, which is often associated with malignant arrhythmias^4–6^. We previously demonstrated in a mouse model of coxsackievirus B3-induced myocarditis that endurance exercise led to more extensive perivascular and interstitial fibrosis, along with a trend toward increased arrhythmogenesis^11^. In this study, we hypothesized that NSAIDs might synergistically exacerbate this remodeling when combined with endurance exercise, drawing on previous experimental findings in viral myocarditis and the prevalent (over)use of these drugs among athletes^13,15^. However, despite an upregulation of collagen genes (COL1A1 and COL3A1), our results did not show significant increases in myocardial fibrosis or scarring in the exercise group receiving NSAIDs. Moreover, the severity of late-stage myocardial inflammation remained comparable across all experimental groups. Notably, ibuprofen administration in exercising mice resulted in improved long-term weight recovery and was associated with a trend toward fewer ventricular arrhythmias in the long term.

Our study is the first to evaluate the impact of NSAIDs (specifically ibuprofen) on long-term outcomes in viral myocarditis. The notion that these anti-inflammatory drugs might exacerbate myocardial injury is informed by a series of mouse studies conducted in the 1980s and early 1990s^20–24^. These studies reported significant increases in cardiomyocyte inflammation, necrosis, and mortality following NSAID administration (including ibuprofen, indomethacin, and sodium salicylate) during the acute and subacute phases of coxsackievirus-induced myocarditis (see Supplementary Fig. 4 and Supplementary Table 1 for an overview)^15^. The authors speculated that these adverse outcomes might result from an exaggerated cytotoxic immune response, impaired viral clearance, or coronary artery vasospasm^15,25^. However, no follow-up research has explored these underlying mechanisms. As noted above, our study did not confirm these prior findings. It is important to note that these earlier studies involved neonatal and very young mice (aged 2 days to 3 weeks), complicating direct comparisons with our findings in adult mice (aged > 15 weeks). Furthermore, our study is the first to utilize the C57BL/6J strain, which is known to exhibit more self-limiting and controlled cardiac damage during viral myocarditis compared to the BALB/c and CD-1 strains previously studied^18,26,27^. We deliberately chose this ‘resistant’ model because it likely reflects the majority of human cases, where myocarditis remains subclinical and resolves completely^28^. Notably, a study by Zhang et al. in a rat model of autoimmune myocarditis found no harmful effects associated with aspirin treatment^29^. Similarly, patient studies did not report adverse clinical outcomes associated with NSAIDs use in acute myo(peri)carditis (see Supplementary Fig. 5)^30–34^. Our findings contribute to the ongoing debate regarding the potential harms of NSAID use in myocarditis and highlight the need for further investigation in both experimental and clinical contexts.

NSAIDs carry a variety of additional risks that necessitate careful consideration^14,35^. Athletes, in particular, may be susceptible to common side effects or may encounter complications specific to their physical demands. These include, among others, irritation of the gastrointestinal tract (potentially exacerbated by sports nutrition and blood flow redistribution during exercise), kidney injury (especially in circumstances of hypovolemia due to dehydration, as may occur with excessive fluid loss during training), delayed healing of musculoskeletal injuries, and blunted training adaptations^14,36,37^. Given these risks, athletes should use these medications cautiously, weighing their benefits against the potential for adverse health outcomes. In this context, it is also important to consider the potential impact of ibuprofen on muscle recovery, particularly through its modulation of satellite cell activity. Satellite cells play a crucial role in skeletal muscle adaptation by supplying new myonuclei and facilitating the repair of damaged muscle fibers^38^. Human studies have shown that NSAID administration (e.g., indomethacin) can suppress the typical exercise-induced increase in satellite cell activity in both untrained individuals and endurance-trained athletes^39,40^. However, the specific effects of ibuprofen in this study—particularly in relation to training initiation, adherence, and recovery in our mouse model with a history of myocarditis—remain unclear.

We observed that NSAIDs and/or exercise reduced initial weight loss, while continued exercise combined with NSAIDs improved body weight recovery. A similar effect of exercise on body weight was noted in our previous study, which was conducted under comparable experimental conditions (i.e., the same mouse strain, virus, exercise duration and intensity, and time of sacrifice)^11^. Notably, both the NSAID and exercise groups displayed a tendency toward more extensive long-term fibrosis, moreover aligning with our prior work on the impact of exercise in modulating fibrosis^11^. Interestingly, NSAID-administered groups showed a trend toward lower (rather than higher) arrhythmogenicity with no identified underlying mechanisms, and there was no significant difference in mortality associated with NSAID use. NSAID administration was discontinued in the mice 16 to 19 days prior to sacrifice, effectively eliminating any acute effects of ibuprofen at the time of harvest. These preliminary findings warrant further investigation; if NSAIDs can indeed reduce the arrhythmogenic potential of myocarditis in athletes, this could be of importance for clinical guidance. Nevertheless, it would be prudent to avoid vigorous exercise during both the acute phase of myocarditis—due to the associated proarrhythmic risk^41^—and the healing phase, given the risk of promoting arrhythmogenic fibrosis. While these findings are intriguing, it is important to acknowledge that translating results from animal models to humans is inherently complex and presents significant challenges. Therefore, these results should be interpreted with caution, and further validation through additional animal studies and, ideally, clinical trials involving athletes, is essential.

### Study limitations

This study exclusively utilized male mice, as epidemiological data and prior research have shown sex differences in viral myocarditis, with males exhibiting greater susceptibility^18,42–44^. We also aimed to avoid potential confounding effects related to the estrous cycle in female mice^45,46^.

The premature death of some mice may have introduced survivorship bias, potentially omitting the most severe myocarditis cases. However, mortality rates were low and similar across the groups. In addition, both this study and our previous research indicate that the degree of myocardial injury in these prematurely deceased animals was comparable to that of those sacrificed at the end of the study^11,18^.

Due to practical and time constraints, in vivo electrophysiology testing was conducted on a randomly selected subset of animals from three out of four experimental groups. Larger sample sizes might have yielded additional insights. Furthermore, while echocardiography could have offered valuable insights into the cardiac impact of our interventions, the virally infected state of the mice prevented us from obtaining these measurements in our viral facilities. Lastly, while our experimental design intentionally focused on the interaction between NSAIDs, endurance exercise, and viral myocarditis, we acknowledge that including additional control groups—particularly uninfected cohorts receiving NSAID treatment, with and without endurance exercise—would have provided insights into the (isolated) cardiac effects of NSAID use.

Forced treadmill running is often employed in murine studies due to its precise control over exercise parameters. However, it is important to note that our study focused on a single type of exercise training; variations in training characteristics (e.g., modality, intensity, duration, frequency) could lead to different outcomes.

## Conclusion

Ibuprofen administration in exercising mice with viral myocarditis resulted in faster weight loss recovery, with no significant differences in residual inflammation and fibrosis after six weeks compared to the exercise-only group, along with a trend toward reduced arrhythmogenesis. Whether this could offer potential benefits for clinical guidance in athletes needs to be confirmed through additional animal studies and clinical trials.

## Supplementary Information

Below is the link to the electronic supplementary material.

Supplementary Material 1

Supplementary Material 2

## Data Availability

The data underlying this article will be shared upon reasonable request to the corresponding author.

## Abbreviations

CDK1NB: Cyclin-dependent kinase inhibitor 1b
CMR: Cardiac magnetic resonance
COL1A1: Collagen type I alpha 1 chain
COL3A1: Collagen type III alpha 1 chain
DMSO: Dimethyl sulfoxide
dPCR: Digital polymerase chain reaction
GAPDH: Glyceraldehyde-3-phosphate dehydrogenase
HE: Hematoxylin and eosin
NSAIDs: Non-steroidal anti-inflammatory drugs
NSVT: Non-sustained ventricular tachycardia
PBS: Phosphate-buffered saline
PEG300: Polyethylene glycol 300
PSR: Picrosirius red
qPCR: Quantitative polymerase chain reaction
SERPINA3N: Serine protease inhibitor A3N
TNFα: Tumor necrosis factor alpha
VERP: Ventricular effective refractory period
VT: Ventricular tachycardia

## References

[1] Liu, T. et al. Current Understanding of the pathophysiology of myocardial fibrosis and its quantitative assessment in heart failure. *Front. Physiol.* **8**, 238 (2017). 28484397 10.3389/fphys.2017.00238 PMC5402617

[2] Zorzi, A. et al. Nonischemic left ventricular Scar as a substrate of Life-Threatening ventricular arrhythmias and sudden cardiac death in competitive athletes. *Circ. Arrhythm. Electrophysiol.* **9** (7), e004229 (2016). 27390211 10.1161/CIRCEP.116.004229 PMC4956679

[3] Shanbhag, S. M. et al. Prevalence and prognosis of ischaemic and non-ischaemic myocardial fibrosis in older adults. *Eur. Heart J.* **40** (6), 529–538 (2019). 30445559 10.1093/eurheartj/ehy713 PMC6657269

[4] Allwood, R. P., Papadakis, M. & Androulakis, E. Myocardial fibrosis in young and veteran athletes: evidence from a systematic review of the current literature. *J. Clin. Med.* **13** (15), 4536 (2024). 39124802 10.3390/jcm13154536 PMC11313657

[5] Malek, L. A. & Bucciarelli-Ducci, C. Myocardial fibrosis in athletes-Current perspective. *Clin. Cardiol.* **43** (8), 882–888 (2020). 32189357 10.1002/clc.23360 PMC7403702

[6] van de Schoor, F. R. et al. Myocardial fibrosis in athletes. *Mayo Clin. Proc.* **91** (11), 1617–1631 (2016). 10.1016/j.mayocp.2016.07.012 27720455

[7] Schnell, F. et al. Subepicardial delayed gadolinium enhancement in asymptomatic athletes: let sleeping dogs lie? *Br. J. Sports Med.* **50** (2), 111–117 (2016). 26224114 10.1136/bjsports-2014-094546

[8] Javed, W., Malhotra, A. & Swoboda, P. Cardiac magnetic resonance assessment of athletic myocardial fibrosis; benign bystander or malignant marker? *Int. J. Cardiol.* **394**, 131382 (2024). 37741350 10.1016/j.ijcard.2023.131382

[9] Fung, G., Luo, H., Qiu, Y., Yang, D. & McManus, B. *Myocarditis Circ. Res.* **118**(3), 496–514 (2016). 26846643 10.1161/CIRCRESAHA.115.306573

[10] McCarthy, R. E. et al. Long-term outcome of fulminant myocarditis as compared with acute (nonfulminant) myocarditis. *N Engl. J. Med.* **342** (10), 690–695 (2000). 10706898 10.1056/NEJM200003093421003

[11] Favere, K. et al. The influence of endurance exercise training on myocardial fibrosis and arrhythmogenesis in a coxsackievirus B3 myocarditis mouse model. *Sci. Rep.* **14** (1), 12653 (2024). 38825590 10.1038/s41598-024-61874-x PMC11144711

[12] Rudgard, W. E., Hirsch, C. A., Rosenbloom, C. & Cox, A. R. Amateur endurance athletes’ use of non-steroidal anti-inflammatory drugs: a cross-sectional survey. *Int. J. Pharm. Pract.* **27** (1), 105–107 (2019). 30019790 10.1111/ijpp.12469

[13] Lippi, G., Franchini, M., Guidi, G. C. & Kean, W. F. Non-steroidal anti-inflammatory drugs in athletes. *Br. J. Sports Med.* **40** (8), 661–662 (2006). discussion 2–3. 16864562 10.1136/bjsm.2006.027342 PMC2579445

[14] Warden, S. J. Prophylactic use of NSAIDs by athletes: a risk/benefit assessment. *Phys. Sportsmed.* **38** (1), 132–138 (2010). 20424410 10.3810/psm.2010.04.1770

[15] Meune, C., Spaulding, C., Mahe, I., Lebon, P. & Bergmann, J. F. Risks versus benefits of NSAIDs including aspirin in myocarditis: a review of the evidence from animal studies. *Drug Saf.* **26** (13), 975–981 (2003). 14583071 10.2165/00002018-200326130-00005

[16] Favere, K. et al. Cardiac electrophysiology studies in mice via the transjugular route: a comprehensive practical guide. *Am. J. Physiol. Heart Circ. Physiol.* **323** (4), H763–H73 (2022). 36018757 10.1152/ajpheart.00337.2022

[17] Yilmaz, B. & Erdem, A. F. Determination of ibuprofen in human plasma and urine by gas chromatography/mass spectrometry. *J. AOAC Int.* **97** (2), 415–420 (2014). 24830154 10.5740/jaoacint.11-414

[18] Favere, K. et al. The natural history of CVB3 myocarditis in C57BL/6J mice: an extended in-depth characterization. *Cardiovasc. Pathol.* 107652. (2024). 10.1016/j.carpath.2024.107652 38750778

[19] Schefer, V. & Talan, M. I. Oxygen consumption in adult and AGED C57BL/6J mice during acute treadmill exercise of different intensity. *Exp. Gerontol.* **31** (3), 387–392 (1996). 9415121 10.1016/0531-5565(95)02032-2

[20] Costanzo-Nordin, M. R., Reap, E. A., O’Connell, J. B., Robinson, J. A. & Scanlon, P. J. A nonsteroid anti-inflammatory drug exacerbates Coxsackie B3 murine myocarditis. *J. Am. Coll. Cardiol.* **6** (5), 1078–1082 (1985). 2995470 10.1016/s0735-1097(85)80312-0

[21] Rezkalla, S., Khatib, G. & Khatib, R. Coxsackievirus B3 murine myocarditis: deleterious effects of nonsteroidal anti-inflammatory agents. *J. Lab. Clin. Med.* **107** (4), 393–395 (1986). 2420912

[22] Rezkalla, S. et al. Effect of indomethacin in the late phase of coxsackievirus myocarditis in a murine model. *J. Lab. Clin. Med.* **112** (1), 118–121 (1988). 2839587

[23] Khatib, R., Reyes, M. P., Smith, F., Khatib, G. & Rezkalla, S. Enhancement of coxsackievirus B4 virulence by indomethacin. *J. Lab. Clin. Med.* **116** (1), 116–120 (1990). 1695914

[24] Khatib, R. et al. Focal ventricular thinning caused by indomethacin in the late phase of coxsackievirus B4 murine myocarditis. *Am. J. Med. Sci.* **303** (2), 95–98 (1992). 1311498 10.1097/00000441-199202000-00006

[25] Bryson, T. D. & Harding, P. Prostaglandin E2 and myocarditis; friend or foe? *Biochem. Pharmacol.* **217**, 115813 (2023). 37722627 10.1016/j.bcp.2023.115813

[26] Fairweather, D. & Rose, N. R. Coxsackievirus-induced myocarditis in mice: a model of autoimmune disease for studying immunotoxicity. *Methods* **41** (1), 118–122 (2007). 17161308 10.1016/j.ymeth.2006.07.009 PMC1764911

[27] Corsten, M. F., Schroen, B. & Heymans, S. Inflammation in viral myocarditis: friend or foe? *Trends Mol. Med.* **18** (7), 426–437 (2012). 22726657 10.1016/j.molmed.2012.05.005

[28] Daniels, C. J. et al. Prevalence of clinical and subclinical myocarditis in competitive athletes with recent SARS-CoV-2 infection: results from the big ten COVID-19 cardiac registry. *JAMA Cardiol.* **6** (9), 1078–1087 (2021). 34042947 10.1001/jamacardio.2021.2065 PMC8160916

[29] Zhang, S. et al. Effects of cyclosporine, prednisolone and aspirin on rat autoimmune giant cell myocarditis. *J. Am. Coll. Cardiol.* **21** (5), 1254–1260 (1993). 8459085 10.1016/0735-1097(93)90254-x

[30] Buiatti, A. et al. Clinical presentation and long-term follow-up of perimyocarditis. *J. Cardiovasc. Med. (Hagerstown)*. **14** (3), 235–241 (2013). 22644404 10.2459/JCM.0b013e328351da6e

[31] Imazio, M. et al. Good prognosis for pericarditis with and without myocardial involvement: results from a multicenter, prospective cohort study. *Circulation* **128** (1), 42–49 (2013). 23709669 10.1161/CIRCULATIONAHA.113.001531

[32] Ammirati, E. et al. Clinical presentation and outcome in a contemporary cohort of patients with acute myocarditis: multicenter Lombardy registry. *Circulation* **138** (11), 1088–1099 (2018). 29764898 10.1161/CIRCULATIONAHA.118.035319

[33] Berg, J. et al. Non-steroidal anti-inflammatory drug use in acute myopericarditis: 12-month clinical follow-up. *Open. Heart*. **6** (1), e000990 (2019). 31168382 10.1136/openhrt-2018-000990 PMC6519432

[34] Mirna, M. et al. Treatment with Non-Steroidal Anti-Inflammatory drugs (NSAIDs) does not affect outcome in patients with acute myocarditis or myopericarditis. *J. Cardiovasc. Dev. Dis.* ;**9**(2). (2022). 10.3390/jcdd9020032 PMC8880264 35200686

[35] Pham, H. & Spaniol, F. The efficacy of Non-Steroidal Anti-Inflammatory drugs in athletes for injury management, training response, and athletic performance: A systematic review. *Sports (Basel)* ;**12**(11). (2024). 10.3390/sports12110302 PMC11598303 39590904

[36] Fitzpatrick, D., Leckie, T., Heine, G. & Hodgson, L. The use of pain killers (NSAIDs) in athletes: how large is the risk? *J. Sci. Med. Sport*. **28** (3), 198–205 (2025). 39665963 10.1016/j.jsams.2024.11.010

[37] Adami, P. E. et al. Cardiovascular effects of doping substances, commonly prescribed medications and ergogenic aids in relation to sports: a position statement of the sport cardiology and exercise nucleus of the European association of preventive cardiology. *Eur. J. Prev. Cardiol.* **29** (3), 559–575 (2022). 35081615 10.1093/eurjpc/zwab198

[38] Hawke, T. J. Muscle stem cells and exercise training. *Exerc. Sport Sci. Rev.* **33** (2), 63–68 (2005). 15821426 10.1097/00003677-200504000-00002

[39] Mackey, A. L. et al. The influence of anti-inflammatory medication on exercise-induced myogenic precursor cell responses in humans. *J. Appl. Physiol. (1985)*. **103** (2), 425–431 (2007). 17463304 10.1152/japplphysiol.00157.2007

[40] Mikkelsen, U. R. et al. Local NSAID infusion inhibits satellite cell proliferation in human skeletal muscle after eccentric exercise. *J. Appl. Physiol. (1985)*. **107** (5), 1600–1611 (2009). 19713429 10.1152/japplphysiol.00707.2009 PMC3774508

[41] Pelliccia, A. et al. 2020 ESC guidelines on sports cardiology and exercise in patients with cardiovascular disease: the task force on sports cardiology and exercise in patients with cardiovascular disease of the European society of cardiology (ESC). *Eur. Heart J.* **42** (1), 17–96 (2020). 10.1093/eurheartj/ehaa605 32860412

[42] Cocker, M. S., Abdel-Aty, H., Strohm, O. & Friedrich, M. G. Age and gender effects on the extent of myocardial involvement in acute myocarditis: a cardiovascular magnetic resonance study. *Heart* **95** (23), 1925–1930 (2009). 19710029 10.1136/hrt.2008.164061

[43] Fairweather, D. et al. Sex and gender differences in myocarditis and dilated cardiomyopathy: an update. *Front. Cardiovasc. Med.* **10**, 1129348 (2023). 36937911 10.3389/fcvm.2023.1129348 PMC10017519

[44] Caforio, A. L. et al. A prospective study of biopsy-proven myocarditis: prognostic relevance of clinical and aetiopathogenetic features at diagnosis. *Eur. Heart J.* **28** (11), 1326–1333 (2007). 17493945 10.1093/eurheartj/ehm076

[45] Aguiar, A. S. Jr., Speck, A. E., Amaral, I. M., Canas, P. M. & Cunha, R. A. The exercise sex gap and the impact of the estrous cycle on exercise performance in mice. *Sci. Rep.* **8** (1), 10742 (2018). 30013130 10.1038/s41598-018-29050-0 PMC6048134

[46] Klein, S. L. & Flanagan, K. L. Sex differences in immune responses. *Nat. Rev. Immunol.* **16** (10), 626–638 (2016). 27546235 10.1038/nri.2016.90

