# Supplementary material for: Impact of NSAIDs and endurance exercise on myocardial fibrosis and arrhythmogenesis in murine coxsackieviral myocarditis
Source: Sci Rep. 2025 Aug 12;15:29607. doi: 10.1038/s41598-025-13437-x (PMC12343846; doi:10.1038/s41598-025-13437-x)
Supplement: Supplementary file 2 — Supplementary Material 2 [file 41598_2025_13437_MOESM2_ESM.docx]

**Supplementary figures and supplementary figure legends**

Supplementary Figure 1. Experimental study design.


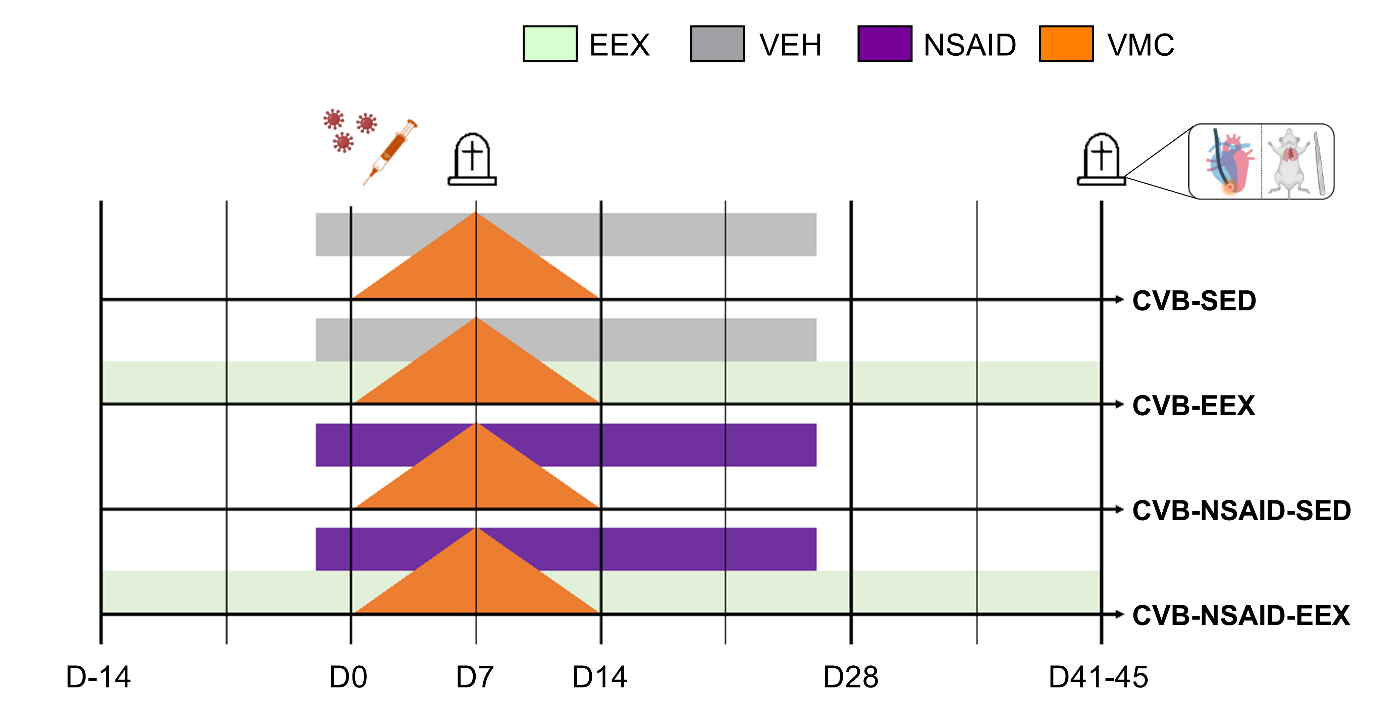


Supplementary Figure 1. Experimental study design. Schematic overview of the study design. Mice were randomly assigned to four experimental groups. The exercise groups (EEX) underwent daily forced treadmill running throughout the study period. All mice received an intraperitoneal inoculation of the human coxsackievirus B3 (CVB) strain Nancy at D0 to induce self-limiting viral myocarditis (VMC). Prior to viral inoculation (D-3), osmotic mini-pumps were subcutaneously implanted to ensure continuous delivery of ibuprofen (NSAID) or a vehicle control. At the chronic disease phase, immediately prior to sacrifice, electrophysiology studies were conducted on a subset of animals to assess ventricular arrhythmogenicity.

Supplementary Figure 2. Exercise training effects.


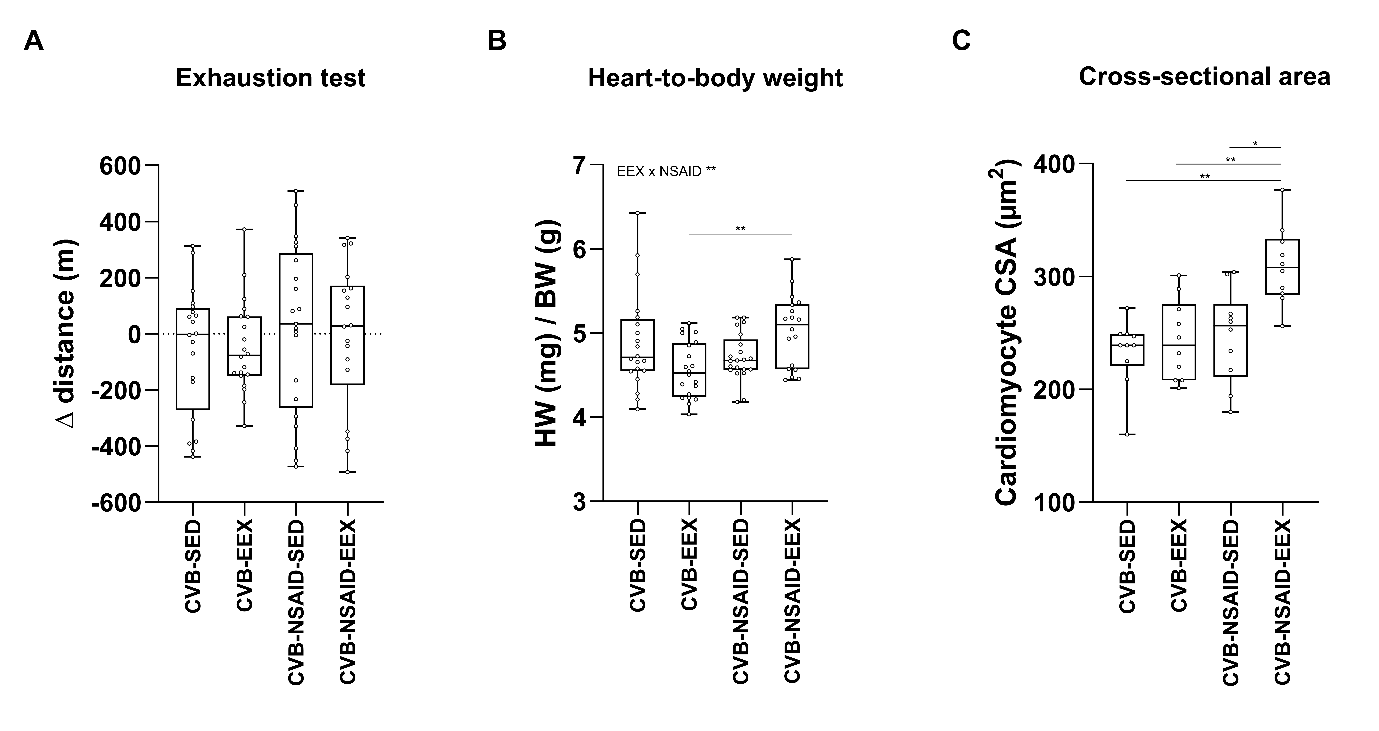


Supplementary Figure 2. Exercise training effects. **A**, Treadmill exhaustion testing. The Y-axis indicates the difference in exercise capacity between testing at the study end and at baseline. No significant differences in exercise capacity were observed between groups. **B**-**C**, Exercise-induced cardiac hypertrophy as evaluated by (**B**) heart-to-body weight ratios and (**C**) cardiomyocyte cross-sectional area. **A**-**B**, Two-way ANOVA with Tukey’s post-hoc test. **C**, Kruskal-Wallis *H* testing with Dunn’s multiple comparisons. Group sizes for panels **A**-**C**: CVB-SED: *n* = 10-20, CVB-EEX: *n* = 10-20, CVB-NSAID-SED: *n* = 10-21, CVB-NSAID-EEX: *n* = 10-18.

Supplementary Figure 3. Myocardial inflammation during the acute disease phase


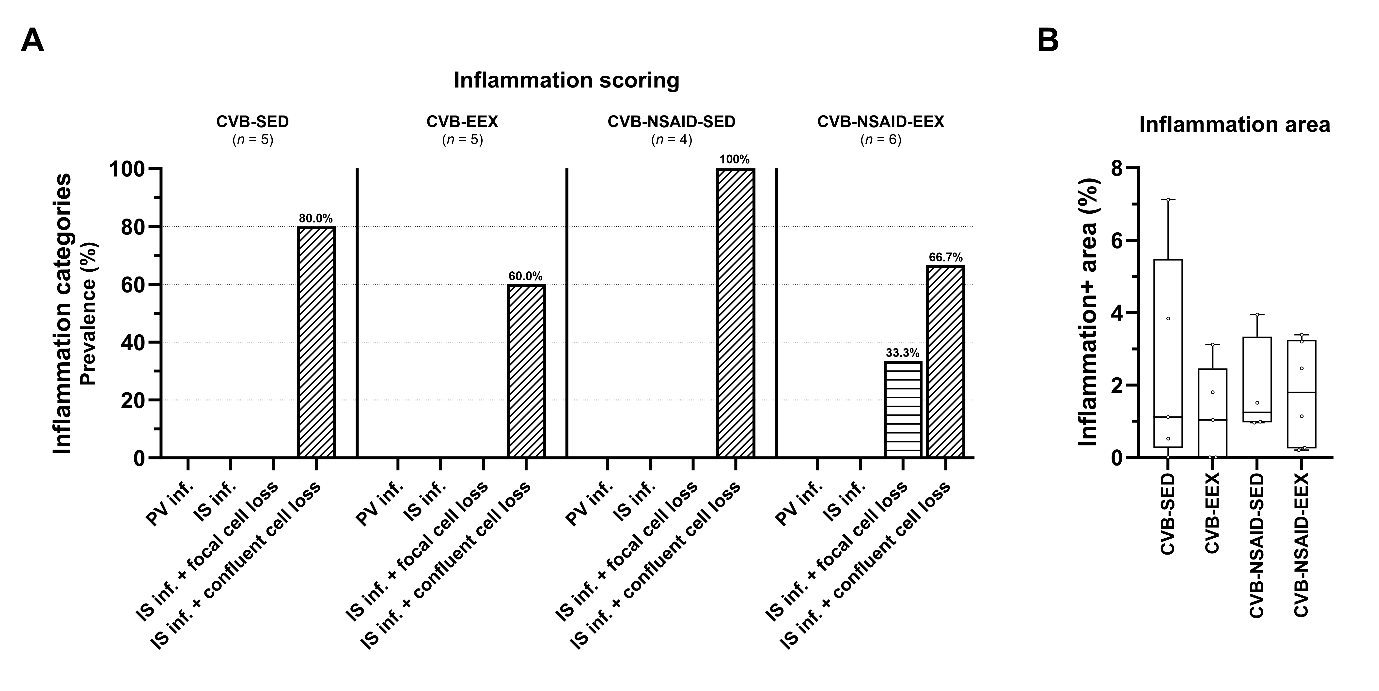


Supplementary Figure 3. Myocardial inflammation during the acute phase. **A**, Distribution of myocardial injury scores. **B**, Quantitative assessment of the area of inflammation in the myocardium. The percentage of inflammation area did not differ significantly among groups (*P*=.797). Kruskal-Wallis *H* test. Data are represented as (**A**) frequency histograms or (**B**) boxplots with interquartile ranges. Group sizes for panels **A**-**B**: CVB-SED: *n* = 5, CVB-EEX: *n* = 5, CVB-NSAID-SED: *n* = 4, CVB-NSAID-EEX: *n* = 6.

Supplementary Figure 4. Viral myocarditis and NSAIDs: current evidence from mice studies.


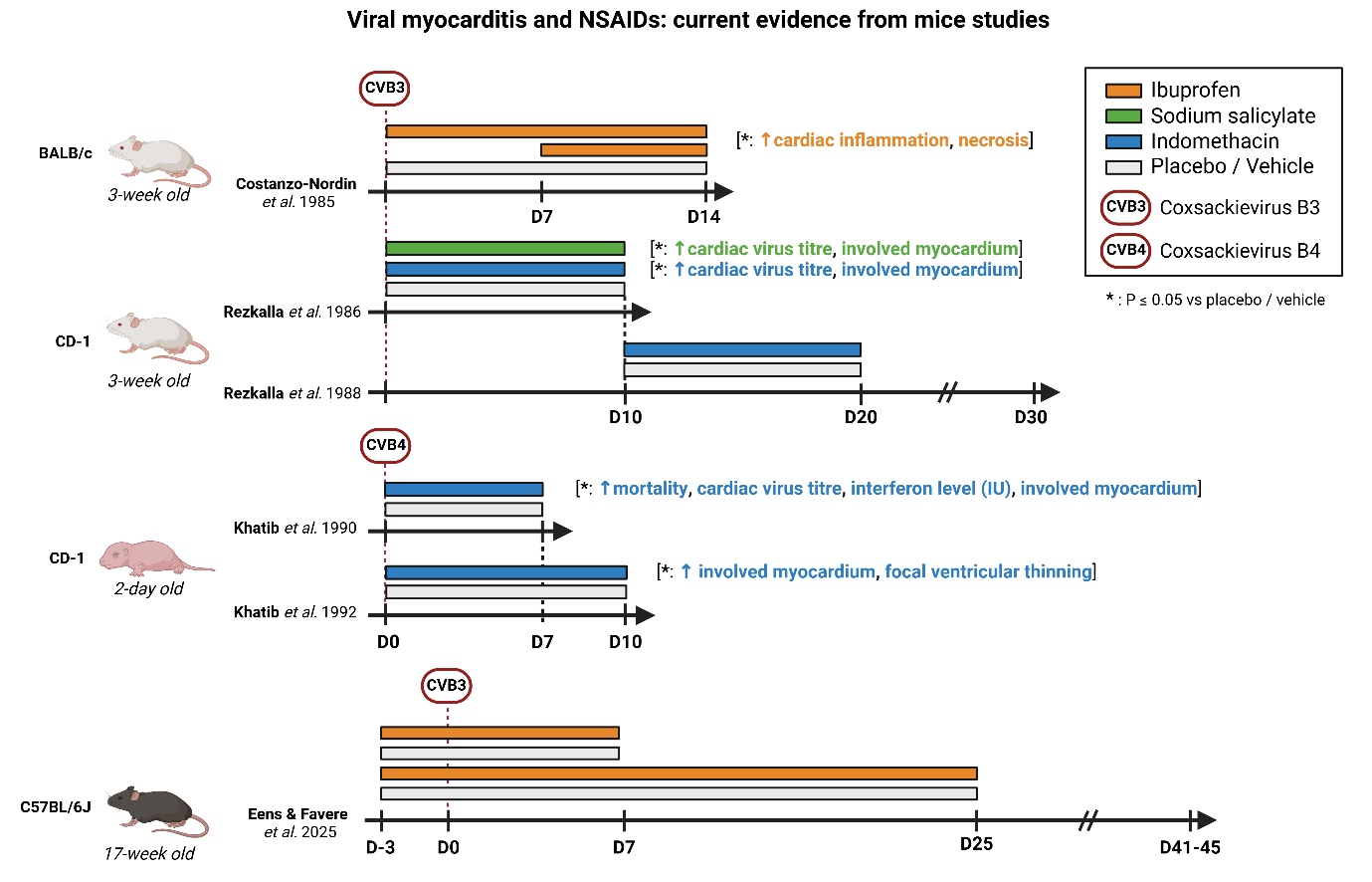


Supplementary Figure 4. Viral myocarditis and NSAIDs: current evidence from mice studies. Schematic overview of historical mouse studies investigating the interplay between NSAIDs and coxsackievirus-induced myocarditis.

Supplementary Figure 5. Myocarditis and NSAIDs: current evidence in humans.


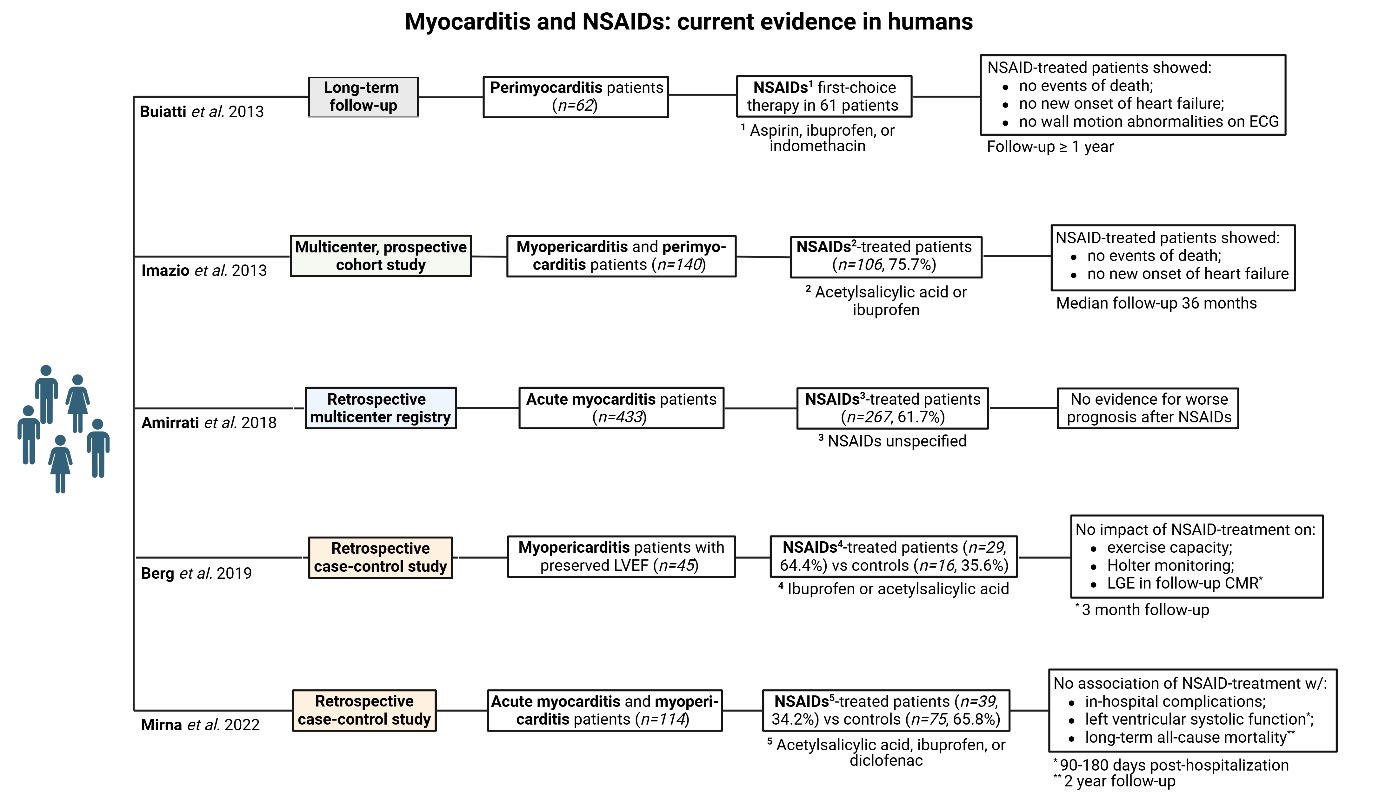


Supplementary Figure 5. Myocarditis and NSAIDs: current evidence in humans. Schematic overview of evidence from studies on the effects of NSAID treatment in patients with acute myocarditis, myopericarditis, and perimyocarditis.

**Supplementary Tables**

Supplementary Table 1. Viral myocarditis and NSAIDs: current evidence from mice studies.

| First Author, Year (Ref) | Virus – Route – Dose | Mice (Age) | Drugs | | Viral load | | Histopathology | | Cardiac function | Mortality |
| --- | --- | --- | --- | --- | --- | --- | --- | --- | --- | --- |
|  |  |  | Intervention | Control | Myocardium | Serum | Inflammation | Fibrosis |  |  |
| Costanzo-Nordin, 1985 (20) | CVB3 (Nancy strain) – IP – 1.75 x 10^7^ | BALB/c (3 weeks old) | Ibuprofen (15 mg/kg) IP – daily – D1-D14 | Diluent – IP – daily – D1-D14 | D7: negative D14: negative |  | D7: inflammation 1.8 vs. 1.6 (NS); necrosis 1.4 vs 1.2 (NS) D14: inflammation 3.1 vs 2.1 (P<0.05); necrosis 3.0 vs. 1.5 (P<0.05); severe inflammation (score 3) 9/12 (75%) vs. 2/10 (20%); severe widespread inflammation (score 4) 3/12 (25%) vs. 0/10 (0%); severe necrosis 8/12 (66%) vs. 1/10 (10%); severe widespread necrosis (score 4) 4/12 (33%) vs. 0/10 (0%); dystrophic calcification and dense bands of mononuclear infiltrates vs. Less calcification and thin bands of mononuclear infiltrates |  |  |  |
|  |  |  | Ibuprofen (15 mg/kg) IP – daily – D7-D14 |  | D7: negative D14: negative |  | D14: inflammation 2.9 vs 2.1 (P<0.05); necrosis 2.7 vs. 1.5 (P<0.05); severe inflammation (score 3) 10/14 (71%) vs. 2/10 (20%); severe widespread inflammation (score 4) 4/14 (28%) vs. 0/10 (0%); severe necrosis 9/14 (61%) vs. 1/10 (10%); severe widespread necrosis (score 4) 4/14 (28%) vs. 0/10 (0%); dystrophic calcification and dense bands of mononuclear infiltrates vs. Less calcification and thin bands of mononuclear infiltrates |  |  |  |
| Rezkella, 1986 (21) | CVB3 (Nancy strain) – IP – 3 x 10^5^ TCID50 | CD1 (3 weeks) | Sodium salicylate (0.005 mg/gm) – IP – 2/d - D0-D9 | Saline D0-D9 | D3: 5.34 vs. 4.23 (P=0.004) D6: 4.72 vs. 3.4 (P=0.006) D10: 2.12 vs. 1.8 | D3: comparable virus titers in all groups | Severe lesions: 23.3% vs. 3.2% D3: 0.69 vs. 0.08 (NS) D6: 2.11 vs. 1.67 (NS) D10: 2.22 vs. 1.56 (NS) D15: 0.75 vs. 1.25 (NS) |  |  | 2/33 (6%) vs. 0/34 (0%) |
|  |  |  | Indomethacin (0.001 mg/gm) – IP – 2/d – D0-D9 |  | D3: 4.94 vs. 4.23 D6: 4.78 vs. 3.4 (P=0.006) D10: 2.0 vs. 1.8 |  | Severe lesions: 46.7% vs. 3.2% D3: 0.0 vs. 0.08 (NS) D6: 2.7 vs. 1.67 (NS) D10: 1.8 vs. 1.56 (NS) |  |  | 4/15 (27%) vs. 0/34 (0%) |
| Rezkella, 1988 (22) | CVB3 (Nancy strain) – IP – 3 x 10^4^ TCID50 | CD1 (3 weeks) | Indomethacin (0.001 mg/gm) – IP – 2/d – D11-D11-D20 | Saline – IP – 2/d – D11-D20 |  |  | D20: inflammation 1.7 vs. 1.4 (NS) ; necrosis 1.0 vs. 1.7 (P<0.05) ; mineralisation 0/10 vs. 5/10 (P<0.016) D30: inflammation 0.3 vs. 0.5 (NS); necrosis 0.6 vs. 0.6 (NS); mineralisation 2/10 vs. 3/10 (NS) |  |  | 3/20 (15%) vs. 0/20 (0%)(NS) |
| Khatib, 1990 (23) | CVB4 (Dowell strain) – IP – 10^4^ TCID50 | (2 days) | Indomethacin (0.0005 mg/gm) – IP – 2/d – D0-D6 | Saline – IP – 2/d – D0-D6 | D2: 8.08 vs. 7.56 (NS) D4: 5.3 vs. 3.9 (P=0.038) D7: 1.18 vs. 0.4 (P=0.028) |  | D7: myocarditis incidence 5/6 vs. 0/6 ; inflammation 0.8 vs. 0 ; necrosis 0.8 vs. 0 ; mineralisation 0.2 vs. 0 ; myocardium involved 5.8% vs. 0% (P=0.008) D21: myocarditis incidence 6/7 vs. 2/4 ; inflammation 0.71 vs. 0.5 ; necrosis 0.86 vs. 0.5 ; mineralisation 0.14 vs. 0 ; myocardium involved 6.42% vs. 1.25% (P=0.028) |  |  | 22/45 (49%) vs. 7/27 (26%)(NS) |
| Khatib, 1992 (24) | CVB4 – IP – 10^4.2^ TCID50 | CD1 (2 days) | Indomethacin (0.001 mg/kg) – IP – 2/d – D10-D20 | Saline – IP - 2/d – D10-D20 |  |  | Myocarditis: 100% vs. 90% Inflammation index: 0.6 vs. 0.7 (NS) Involved myocardium: 150 vs. 35 (P<0.05) Focal thinning: 5/12 (41.7%) vs. 0% (P<0.05) | Necrosis/ scar index: 0.4 vs. 0.3 (NS) |  | 7/15 (47%) vs. 2/12 (17%)(NS) |
| Data are presented as NSAID group vs. control. - Abbreviations: CVB3, coxsackievirus B3; CVB4, coxsackievirus B4; IP, intraperitoneal; NS, not significant; TCID50, median tissue culture infectious dose. | | | | | | | | | | |
